# An integrated analysis of mRNA and sRNA transcriptional profiles in tomato root: Insights on tomato wilt disease

**DOI:** 10.1371/journal.pone.0206765

**Published:** 2018-11-05

**Authors:** Min Zhao, Hui-Min Ji, Ying Gao, Xin-Xin Cao, Hui-Ying Mao, Shou-Qiang Ouyang, Peng Liu

**Affiliations:** 1 College of Horticulture and Plant Protection, Yangzhou University, Yangzhou, Jiangsu, China; 2 Joint International Research Laboratory of Agriculture and Agri-Product Safety, Yangzhou University, Yangzhou, Jiangsu, China; 3 Texting Center, Yangzhou University, Yangzhou, Jiangsu, China; Huazhong Agriculture University, CHINA

## Abstract

Tomato wilt disease caused by *Fusarium oxysporum* f. sp. *lycopersici* (FOL) is a worldwide destructive disease of tomato. As exploring gene expression and function approaches constitute an initial point for investigating pathogen-host interaction, we performed RNA-seq and sRNA-seq analysis to investigate the transcriptome of tomato root under FOL infection. Differentially expressed (DE) protein-coding gene and miRNA gene profiles upon inoculation with FOL were presented at twenty-four hours post-inoculation in four treatments. A total of more than 182.6 million and 132.2 million high quality clean reads were obtained by RNA-seq and sRNA-seq, respectively. A large overlap was found in DE mRNAs between susceptible cultivar Moneymaker and resistant cultivar Motelle. Gene Ontology terms were mainly classified into catalytic activity, metabolic process and binding. Combining with qRT-PCR and Northern blot, we validated the transcriptional profile of five genes and five miRNAs conferred to FOL infection. Our work allowed comprehensive understanding of different transcriptional reaction of genes/miRNAs between the susceptible and resistant cultivars tomato to the FOL challenge, which could offer us with a future direction to generate models of mediated resistance responses.

## Introduction

*Fusarium oxysporum* f. sp. *lycopersici* (race 2) (as FOL in this study) is a necrotrophic pathogen which is the causal agent of tomato wilt disease worldwide [1, 2]. Under appropriate conditions, FOL infection leads to clogged vessels resulting in yellowing of leaves, wilting and finally death of the whole plant. According to their specific pathogenicity to tomato cultivars, three physiological races of FOL were distinguished [1, 2].

Tomato (*Solanum lycopersicum*) is a worldwide agriculture economic crop and also has been studied as a crucial model plant for studying the genetics and molecular basis of resistance mechanisms. Four plant resistance (*R*) genes have been introgressed from wild tomato species including the *I* (or *I-1*) and *I-2* from *S*. *pimpinellifolium*, and the *I-3* and *I-7* from *S*. *pennellii*. Among these *R* genes, so far, *I-2*, *I-3* and *I-7* have been cloned, encoding an NBS-LRR protein like most known *R* genes [3–6]. Previous works have unveiled that the *I-2* and *I-3* conferred resistance to race 2 and race 3 strains of FOL, respectively [4, 5]. The *I-2* locus encodes an *R* protein that recognizes Avr2 effector protein from FOL (race 2) [7]. The *I-3* encodes an S-receptor-like kinase (SRLK) that confers *Avr3*-dependent resistance to FOL (race 3) [5]. Two near-isogenic tomato cultivars susceptible Moneymaker (*i-2*/*i-2*) and resistant Motelle (*I-2*/*I-2*) were recruited to study the interaction between tomato and FOL [1, 8, 9].

Basically, transcriptome analysis is a very important tool to discover the molecular basis of plant-pathogen interaction globally, allowing dissection of the pattern of pathogen activities and molecular repertoires available for defense responses in host plant. By taking advantage of RNA-seq technology, certain number of transcriptome profiling studies of plants inoculated by *Fusarium* fungus have been presented including banana [10], cabbage [11], watermelon [12], mango [13], and *Arabidopsis* [14, 15].

MicroRNAs (miRNAs) (20–24 nucleotides in length) are derived from endogenous single-stranded non-coding small RNAs with imperfectly base-paired hairpin structures, which regulate gene expression at the post-transcriptional level via base-pairing cleavage, or the translational level in complex with Argonaute proteins by repression the target mRNAs [16–19]. To date, a few of studies have demonstrated that miRNAs play critical roles in various biotic and abiotic stress responses, especially stress responses [20, 21] and innate immunity [22–24]. MiRNAs might confer pathogen response by regulating plant hormonal network, such as auxin, jasmonic acid (JA), ethylene (ET), as well as salicylic acid (SA)-mediated defense. For example, miR393 targets the AFB/TIR1 leading to enhance an accumulation of pathogen-resistant protein [25, 26]. Our previous study showed that two miRNAs, sly-miR482f and sly-miR5300, along with several *NBS-LRR* (or like) genes, were verified to play a crucial role in response to FOL infection in tomato [27, 28]. However, there is far less attention to understand how genes and miRNAs were integrated into the dynamic and complex regulatory network resulting in the enhancement of resistance to FOL in tomato.

Beyond the importance of the tomato wilt disease caused by FOL, the knowledge of tomato reprogramming under the onset of wilt disease still remains unknown. The object of this study is to explore transcriptional changes in Moneymaker (susceptible) and Motelle (resistant) isogenic tomato cultivars infected by FOL. In addition to genes prediction, our results also uncovered a bunch of FOL-responsive miRNAs in tomato for further functional characterization, which would provide a broader view of the dynamics of tomato defense triggered by FOL infection.

## Materials and methods

### Tomato materials and fungal culture

Susceptible cultivar Moneymaker (*i-2*/*i-2*) and resistant cultivar Motelle (*I-2*/*I-2*) were applied for plant infection and libraries construction in this study. Tomato seedlings were grown at 25°C with a 16/8-h light/dark cycle for two weeks. The wild-type FOL (race 2) strain is FGSC 9935 (known as FOL 4287 or NRRL 34936). Tomato seedlings were removed from soil and roots were rinsed with running water gently followed by incubating in the solution of FOL conidia at a concentration of 1x10^8^ conidia/ml for 30 minutes. Water treated tomato seedlings were used as the control. Forty seedlings were used for each treatment. All treated tomato seedlings were then transferred to pots containing vermiculite and placed in a growth chamber at 25°C for 24 hours (16-hour light, 8-hour dark) as descripted previously [25]. After infection, clean roots were collected and immediately frozen in liquid nitrogen for total RNA extraction. In order to minimize experimental variations, all root samples were collected in three independently repeated experiments.

### RNA extraction, library preparation, and sequencing

Total RNA was isolated from roots using TRIzol Reagent (#15596026, Life Technologies, CA, USA) as descripted previously [27]. For each sample, all roots from three biological repeats were pooled together for total RNA extraction.

For mRNA library construction, after the total RNA extraction followed by DNase I treatment, mRNA was enriched by magnetic beads with Oligo (dT). The mRNA was sheared into short fragments in the fragmentation buffer. Then cDNA was synthesized using the mRNA fragments as templates. cDNAs were purified and resolved with elution buffer for end reparation and single nucleotide A (adenine) addition followed by adding adapters to cDNAs. After agarose gel electrophoresis, the suitable cDNAs were selected for the PCR amplification as templates. During the quality control (QC) steps, Agilent 2100 Bioanaylzer and ABI StepOnePlus Real-Time PCR System were used in quantification and qualification of the sample library. The libraries were sequenced using Illumina HiSeqTM 2000.

For sRNA library construction, 1 μg of total RNA were used for small RNA library generation. Briefly, total RNA samples were separated using polyacrylamide gel electrophoresis (PAGE), and cut out between 18 and 30 nt stripe to recover small RNA. 3' and 5' adapter were ligated at both ends, followed by reverse transcribed with Superscript II Reverse transcriptase using adapter-specific RT-primers. PCR products were then gel purified to enrich special fragments. The quality control (QC) steps were described as above. The purified high-quality cDNA library was sequenced using Illumina Genome HiSeq4000.

### Bioinformatics analysis of RNA-seq and sRNA-seq

Primary sequencing data that produced by Illumina HiSeqTM 2000/HiSeq4000, called as raw reads, were subjected to quality control (QC). The QC alignment data was utilized to calculate distribution of reads on reference genes and mapping ratio. After QC, raw reads were filtered into clean reads which were aligned to the reference sequences [29–31].

For RNA-seq, the clean reads were then aligned to the tomato reference genome downloaded from the Sol Genomics Network using Bowtie v0.12.5 [29] and TopHat v2.0.0 [31, 32] with default settings. Transcript abundance was calculated with Cufflinksv0.9.3 [32] based on fragments per kilo base of transcript permillion fragments mapped (FPKM) under default parameters settings. The transcript abundance was calculated for individual sample files followed further merged pairwise for each comparison (FOL treatment versus water treatment for each cultivar) using Cufflinks utility program-Cuffmerge [31]. The pairwise comparisons of gene expression profiles between the two populations were done using the Cuffdiff program of the Cufflinks version 1.3.0 [32]. The genes were considered significantly differentially expressed if Log_2_ FPKM (fold change) was ≥ 1.0 and false discovery rate (FDR, the adjusted P value) was < 0.01. The q-value which was a positive FDR analogue of the p-value was set to < 0.01 [33].

For sRNA-seq, the sequence data were subsequently processed using in-house software tool SeqQC V2.2. House-keeping small RNAs including tRNAs, rRNAs, snoRNAs and snRNAs were removed by blasting in GenBank (http://www.ncbi.nih.gov/Genbank) servers. The trimmed reads were then aligned to the tomato reference genome downloaded from http://www.mirbase.org (miRBase 21.0) and http://www.ncbi.nlm.nih.gov using Bowtie v0.12.5 (http://www.bioinfogp.cnb.csic.es/tools/venny/index.html.) and TopHat v2.0.0 [34] with default settings. To identify known miRNAs, the remaining unique small RNA sequences were then aligned against the miRBase 21.0 allowing maximum one mismatch. After assigning the known miRNA sequences into their respective groups or families, rest of the sequences were checked for novel miRNAs.

### Functional categorization of DEGs

DEGs were functionally categorized online for all pairwise comparisons according to the Munich Information Center for Protein Sequences (MIPS) functional catalogue [34]. The functional categories and subcategories were regarded as enriched in the genome if an enrichment p-value was below <0.05. The Kyoto Encyclopediaof Genes and Genomes (KEGG) pathway analyses were performed using interface on blast2GO (Blast2GO v2.6.0, http://www.blast2go.com/b2ghome) for all DEGs to identify gene enrichment on a specific pathway.

### Gene ontology (GO) and pathway enrichment analysis

Gene Ontology (GO) and pathway enrichment were performed using DAVID software. Graphs of the top 20 enriched GO terms for each library were generated using the Cytoscape Enrichment Map plugin [35, 36].

### Quantitative RT-PCR (qRT-PCR) and Northern blot analysis

Quantitative RT-PCR (qRT-PCR) and Northern blot analysis were performed according to our previous protocol [27]. Briefly again, expression of DEGs were determined using qRT-PCR. cDNAs were generated from 1 μg of total RNA using the SMART MMLV Reverse Transcriptase (Takara, Mountain View, CA) followed by diluting two times and using as template for qRT-PCR, which was performed with the CFX96 real-time PCR system (Bio-Rad, Hercules, California, USA). Primers used for qRT-PCR were designed from 3-UTR for individual gene. Each reaction mixture (20 μL) contained 1 μL of cDNA template, 10 μL of SYBR1 Green PCR Master Mix (Applied Biosystems, Foster, CA) and 1 μL of each primer (10 μM). For each cDNA sample, three replications were performed. The level of 18S rRNA was used as internal control for normalize the expression level of selected genes, and were calculated as the fold change by comparison between in FOL treated and water in treated samples.

To Northern blot analysis, 10 μg total RNA was resolved on urea denaturing polyacrylamide gels (Urea-PAGE). MiRNA-specific oligonucleotide probes were end-labeled using γ-^32^P-ATP (#M0201, New England Biolabs, Ipswich, MA). Gel staining with ethidium bromide was used as the loading control. All blots were imaged by PhosphorImager (GE Life Sciences, Pittsburgh, PA, USA). The images were cropped and adjusted with brightness and contrast in Photoshop CS6 from original digital images.

### Statistical analyses

All data in this study were analyzed with ANOVA program or Student’s t-test analysis using SPSS 11.5 (SPSS Company, Chicago, IL) for statistical analysis.

## Results

### Post inoculation phenotype in Moneymaker and Motelle

**Fig 1:**
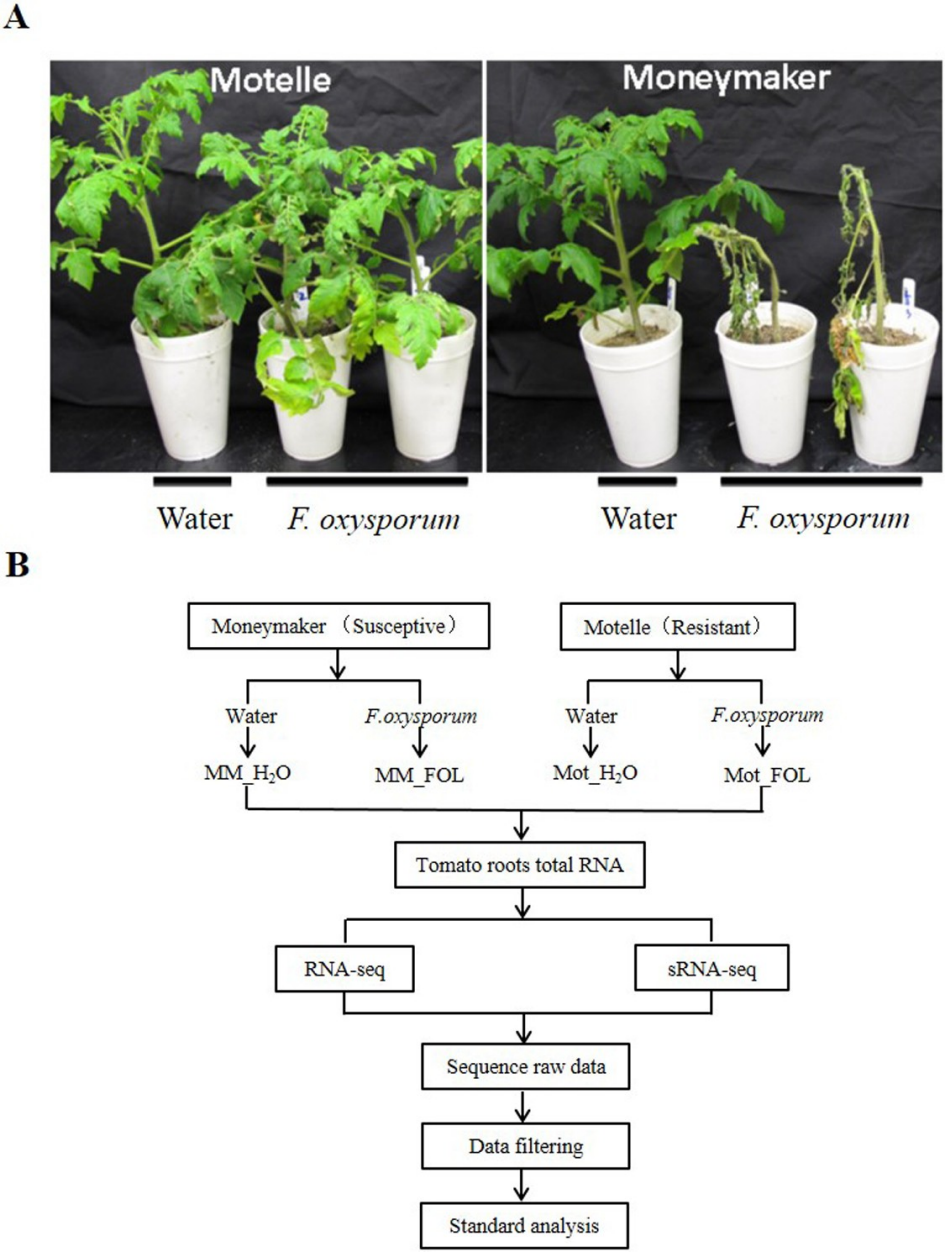
Post inoculation phenotype in susceptible cultivar Moneymaker and resistant cultivar Motelle. A The phenotype of tomato seedlings infected by FOL. Two-week-old tomato seedlings were treated with FOL or water followed by photographing four weeks later. B Briefly mRNA and miRNA detection workflow.

We recruited tomato cultivars susceptible Moneymaker (*i-2*/*i-2*) and resistant Motelle (*I-2*/*I-2*) genotypes. Four weeks after FOL infection, Moneymaker plants exhibited severe wilting symptoms, conversely, Motelle plants displayed strong resistance to FOL infection (Fig 1A). We generated four libraries for RNA-seq and sRNA-seq respectively, including Moneymaker treated with FOL/water (MM_FOL/MM_H_2_O), Motelle treated with FOL/water (Mot_FOL/Mot_H_2_O). Therefore, totally four libraries were constructed. After treating with FOL/water, total RNA from tomato roots were extracted for both RNA-seq and sRNA-seq. A briefly mRNA and miRNA detection workflow was presented to summarize the method utilized in this study (Fig 1B).

### General features of mRNA and miRNA sequencing data mapping and annotation

**Fig 2:**
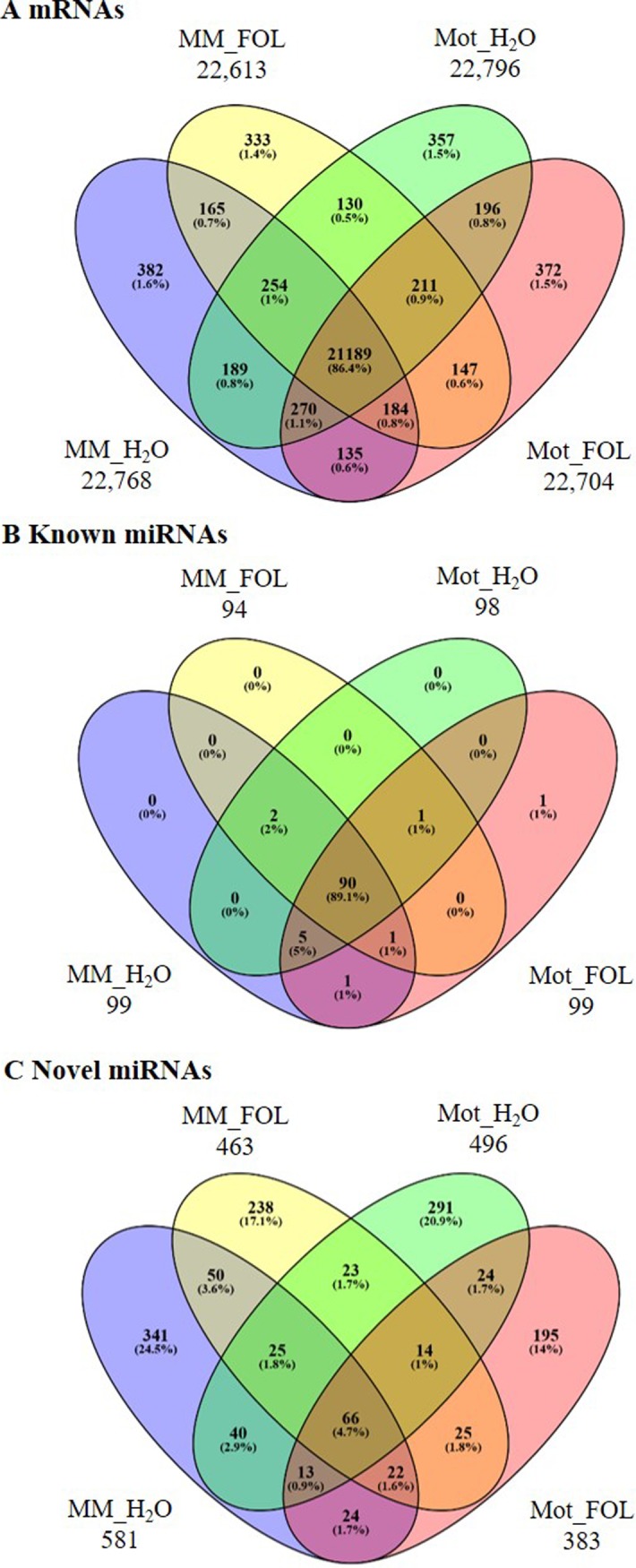
The Venny diagrams showing the overlaps of mRNA/miRNAs among four comparisons of FOL and water treatment. A mRNAs. B Known miRNAs. C Novel miRNAs.

A total of more than 182.6 million high quality clean reads were collected by RNA-seq from four libraries. The sequencing yielded ~46 million clean reads from each library respectively. The clean reads were then bowtied and tophated to the *Solanum lycopersicum* genome using the Tomato Genome assembly (SL3.0) and annotations (ITAG3.0) (www.solgenomics.net/). Approximately three fourths of the total Illumina reads perfectly matched the genome or gene and were used for further analysis. Analysis of the expressed transcripts in the libraries showed that almost equal number of transcripts were observed by ~23 million in four libraries (Table 1). We used Cufflink to measure the expression level of tomato annotated genes. Among these transcripts, 21,189 (86.4%) genes were expressed in all four libraries. Moreover, 382 (1.6%), 333 (1.4%), 357 (1.5%) and 372 (1.5%) genes were uniquely presented in MM_H_2_O, MM_FOL, Mot_H_2_O and Mot_FOL library, respectively (Fig 2A and S1 Table).

**Table 1:** Summary of RNA-seq and sRNA-seq datasets from four libraries.

	Annotation	MM_H_2_O	MM_FOL	Mot_H_2_O	Mot_FOL
RNA-seq	Clean reads	45,616,330	45,635,428	45,680,034	45,661,734
	Genome map rate	75.49%	67.89%	75.87%	70.46%
	Gene map rate	76.87%	68.47%	75.92%	71.26%
	Expressed gene	22,796	22,639	22,825	22,725
	Novel gene	775	761	795	705
	Alternative splicing	32,482	32,689	33,706	32,965
	Total reads apped to genome	75.49%	67.89%	75.87%	70.46%
	Perfect match to genome	63.00%	55.52%	62.61%	57.41%
	Mismatch to genome	12.49%	12.38%	13.26%	13.05%
	Unique match to genome	74.32%	66.85%	74.79%	69.46%
	Total Unmapped reads	24.51%	32.11%	24.13%	29.54%
sRNA-seq	Clean reads	31,192,441	34,969,928	33,000,743	33,125,499
	snRNA	233,962	413,635	360,602	416,831
	rRNA	15,246,656	16,245,295	18,695,564	17,611,875
	snoRNA	88,371	112,863	90,081	138,206
	Repeat	52,8140	318,782	427,681	310,377
	miRNA	339,025	165,398	219,845	143,398
	tRNA	655,213	715,878	642,906	712,388

By sRNA-seq, we collected a total of more than 132.2 million high quality clean reads including 31.2 million from MM_H_2_O, 35.0 million from MM_FOL, 33,0 million from Mot_H_2_O, and 33.1 million from Mot_FOL, respectively. Valid sequences were classified according to the genomic regions matched. The composition of sRNA pool was comprehensive and contained a huge portion of other non-coding RNA species including snRNA, rRNA, snoRNA, repeat, miRNA and tRNA. Of these sRNA, we detected 339,025 miRNAs from MM_H_2_O, 165,398 from MM_FOL, 219,845 from Mot_H_2_O, and 143,398 from Mot_FOL (Tables 1 and S1). Synchronously, a venny diagram was recruited to demonstrate the distribution of miRNAs among the four comparisons. To known miRNAs, we present that the majority of known miRNAs were overlapped among these four libraries (Fig 2B). To novel miRNAs, however, there were 341 (24.5%), 238 (17.1%), 291 (20.9%) and 195 (14%) miRNAs altered the expression uniquely in four libraries respectively (Fig 2C).

### Analysis of differentially expressed (DE) mRNAs and miRNAs and functional classification of DEGs by gene ontology (GO) enrichment analysis

**Fig 3:**
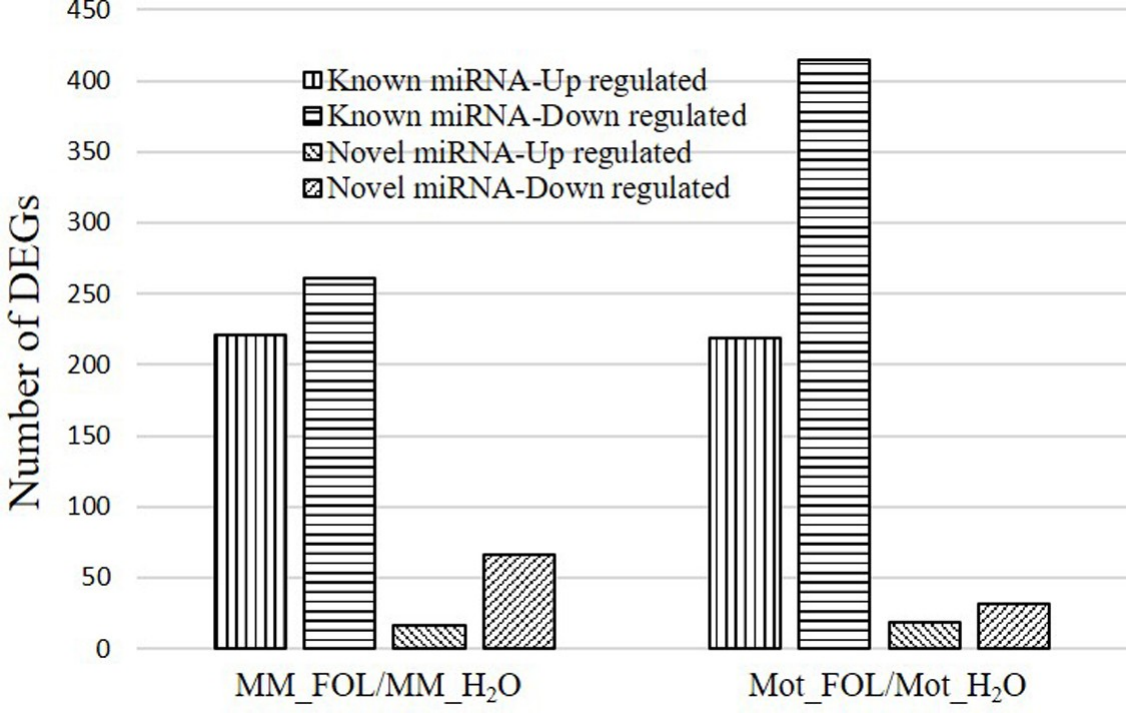
Statistics of differentially expressed miRNAs between FOL and water treatment.

Differentially expressed genes (DEGs) were defined as genes with fold-change > 2 folds and FDR < 0.01. A total number of 3,942 and 4,168 genes were showed significantly differential expression in MM_FOL vs. MM_H_2_O library and Mot_FOL vs. Mot_H_2_O library, respectively. A majority of these DEGs were overlapped in both water and FOL treated two tomato cultivars. Among these DEGs, 221 were down-regulated in MM_FOL vs. MM_H_2_O and 219 were down-regulated in Mot_FOL vs. Mot_H_2_O, while 261 were up-regulated in MM_FOL vs. MM_H_2_O and 415 up-regulated in Mot_FOL vs. Mot_H_2_O (Fig 3, detailed in S1 Table).

Screening of DE miRNAs, the results displayed that the miRNA expression level changed under FOL treatment. The histogram was shown that more miRNAs have altered expression after FOL treatment in Motelle sample (18 up-regulated and 31 down-regulated) compared to in Moneymaker sample (16 up-regulated and 66 down-regulated) (Fig 3). Taken together, FOL treatment had a significant impact on global gene/miRNA expression profile in tomato plants.

**Fig 4:**
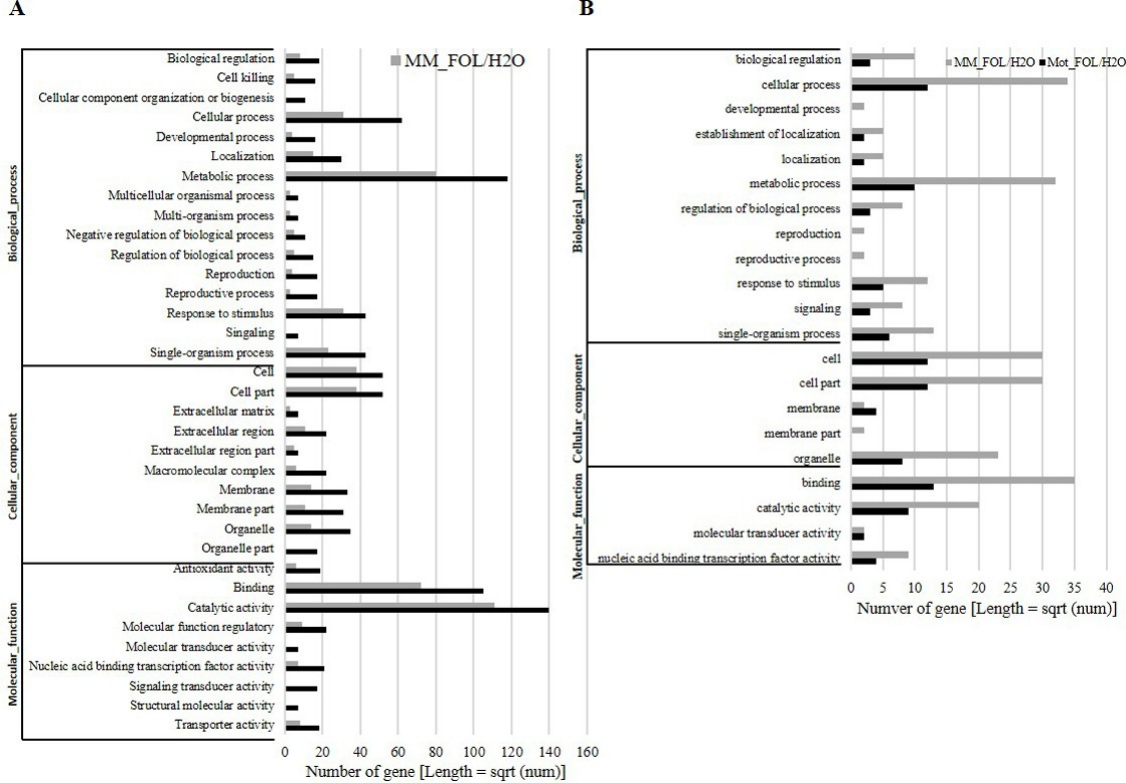
Functional categorization of significantly differentially expressed mRNA and miRNA under FOL invasion in tomato. The results were basically summarized into three main categories: biological processes, cellular components, and molecular functions. All statistically significant genes from four libraries were assigned to GO terms. A mRNA from RNA-seq. B Targets of miRNAs from sRNA-seq.

To explore the distribution of DEGs/DE miRNAs, gene ontology (GO) enrichment analyses were conducted based on these DEGs to elucidate the biological processes/pathways. A total of 530 and 769 GO terms were discovered in MM_FOL vs. MM_H_2_O and Mot_FOL vs. Mot_H_2_O library, respectively, correlated with three main classes: Biological Processes, Cellular Component, and Molecular Function. GO enrichment analysis revealed that GO terms were mainly classified into catalytic activity (104 out of 530 in MM_FOL vs. MM_H_2_O library, and 141 out of 769 in Mot_FOL vs. Mot_H_2_O library) (the same define in the following text), metabolic process (81 out of 530, and 118 out of 769), and binding (72 out of 530, and 104 out of 769). Within the response to stimulus, however, no significant change was presented between these two libraries (31 out of 530, and 36 out of 769) (Fig 4A and S2 Table). To DE miRNAs, cellular process and metabolic process were the two most represented categories in Biological Process, being associated with 23.8% of the coding regions of Moneymaker, and 20.8% of the coding regions of Motelle. The cell and cell part category were associated with 21.7% of the transcripts in Moneymaker, and 22.6% in Motelle, being the two most represented in the Cellular Component class. The majority of the transcripts were found to be annotated with the binding category and catalytic activity in Molecular Function, with 19.9% of the transcripts in Moneymaker and 20.8% in Motelle (Fig 4B and S3 Table). It was worthy to note that when generally compared the two deep sequencing results (RNA-seq and sRNA-seq), we found that there were more regulated transcripts responding to FOL invasion in Motelle than that of in Moneymaker, on the contrary, more regulated miRNAs responding to FOL invasion in Moneymaker than that of in Motelle.

### The profiles and expressions of DEGs in pathogen resistance pathway between susceptible and resistant tomato plants

To further understand the biological functions, the pathway enrichment of DEGs were performed to discover the effect of FOL to host plant. Be worth mentioning, the plant-pathogen pathway was ranked in the 29^th^ (24 out of 356 DEGs) in MM_FOL vs. MM_H_2_O group (Detailed in S4 Table), however, it was presented in the 8^th^ (40 out of 469 DEGs) in Mot_FOL vs. Mot_H_2_O group (Detailed in S5 Table). These results may indicated that more pathogen resistance genes were regulated in resistant cultivar Motelle than that in susceptible cultivar Moneymaker. To summarize, these DEGs included genes encoding WRKY protein (8 genes), receptor kinase (20 genes), MYB transcription factor (7 genes), NBS-ARC protein (18 genes), Calmodulin-like protein (9 genes), MAPK (2 genes) and others (7 genes). Intriguingly, seventeen DEGs were regulated in both Moneymaker and Motelle. (Table 2).

**Table 2:** Detail information of regulated genes involved in the plant-pathogen interaction pathway.

Pathway	Annotation	Target genes involved in the pathway
Plant-pathogen interaction	WRKY transcription factor (8 genes)	Solyc04g072070, **Solyc01g095630**, **Solyc06g068460**, Solyc10g011910, Solyc08g067340, Solyc08g008280, Solyc09g015770, Solyc06g066370.
	Receptor kinase (20 genes)	Solyc06g062450, **Solyc03g059080**, Solyc07g054120, Solyc04g074000, Solyc04g014400, Solyc03g098400, Solyc01g013880, Solyc01g016370, Solyc03g082780, Solyc12g040740, Solyc12g100020, Solyc01g096350, Solyc04g009040, Solyc05g055190, Solyc06g076910, Solyc12g009520, Solyc12g009730, Solyc12g009740, Solyc12g009750, Solyc12g013680.
	MYB transcription factor (7 genes)	**Solyc03g005570**, Solyc10g005460, Solyc12g099120, Solyc12g008670, Solyc04g079360, Solyc11g073120, Solyc02g092930.
	NBS-ARC protein (18 genes)	**Solyc00g174340**, **Solyc09g007020**, Solyc00g174330, Solyc07g006710, Solyc09g007010, Solyc00g102400, Solyc02g084890, Solyc04g009090, Solyc04g009150, Solyc04g026110, Solyc05g008650, Solyc09g098130, Solyc10g047320, Solyc11g020100, Solyc11g069020, Solyc06g048910, Solyc08g007250, Solyc09g098130.
	Calmodulin-like protein (9 genes)	**Solyc11g071750**, **Solyc11g071740**, Solyc03g044900, **Solyc10g006660**, **Solyc02g094000**, **Solyc02g091500**, **Solyc10g006700**, Solyc03g118810, Solyc04g008000.
	MAPK (2 genes)	Solyc05g008020, Solyc06g005170.
	Others (7 genes)	Solyc09g083050, Solyc03g005320, **Solyc05g050350**, **Solyc05g050380**, **Solyc01g094910**, **Solyc11g071760**, **Solyc00g026160**.

Genes (bold) were regulated in both Moneymaker and Motelle. Genes (underlined) were further analyzed by qRT-PCR.

**Fig 5:**
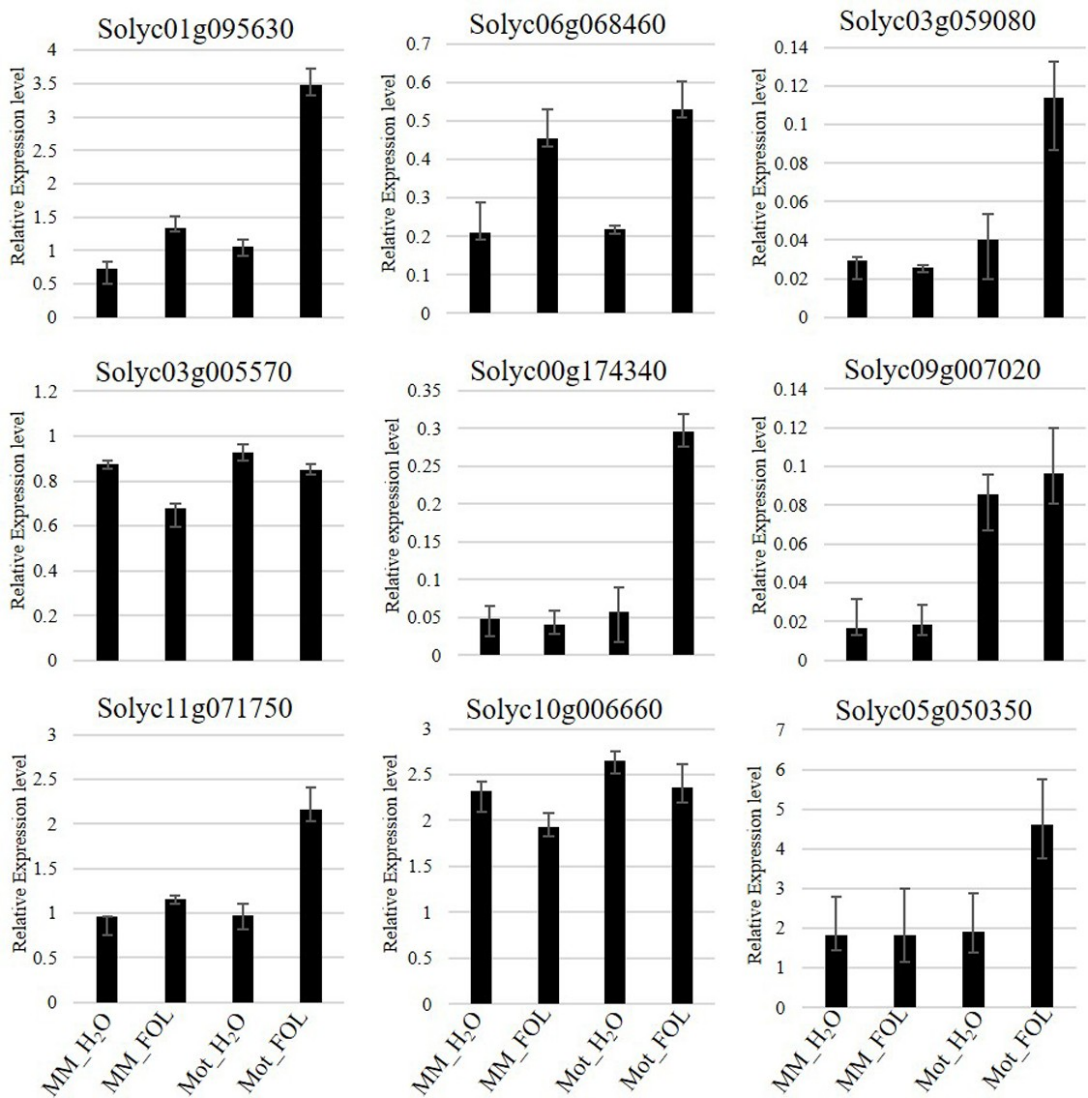
Validation of differentially expressed genes selected in plant-pathogen interaction pathway by qRT-PCR. Total tomato root RNA was reverse transcribed to cDNA used as template for qRT-PCR with gene-specific primers. Each column represents an average of three replicates, and error bars represent the standard error of means.

In the plant-pathogen interaction pathway, nine predicted disease related DEGs, regulated in both Moneymaker and Motelle, were selected to characterize the gene expression profiles by qRT-PCR using primers listed in S8 Table. These DEGs included Solyc01g095630 (SlWRKY41), Solyc06g068460 (SlWRKY40), Solyc03g059080 (Receptor-like serine/threonine protein kinase), Solyc03g005570 (Myb-related transcription factor), Solyc00g174340 (Pathogenesis-related protein 1b), Solyc09g007020 (Pathogenesis-related protein), Solyc11g071750 (Calmodulin-like protein), Solyc10g006660 (Calcium-binding EF hand family protein) and Solyc05g050350 (Cyclic nucleotide gated channel). The results of qRT-PCR showed the similar pattern to sequencing results with minute difference. In detail, Solyc01g095630, Solyc03g059080, Solyc00g174340, Solyc11g071750 and Solyc05g050350 were induced greatly in resistant cultivar Motelle affected by FOL, however, no significant changes were presented in susceptible cultivar Moneymaker between FOL and water treatment. Solyc06g068460 was induced in both Moneymaker and Motelle upon FOL treatment. On the other hand, Solyc11g071750 and Solyc10g006660 were suppressed in both Moneymaker and Motelle plants when treated with FOL (Fig 5).

### MiRNA expression patterns responding to FOL infection and experimental validation of miRNA by Northern blot

We predicted the targets of known and novel miRNAs with the number 1179 and 2615, respectively (S6 Table). In total, thirty-two miRNA families were detected in all libraries in both Moneymaker and Motelle under FOL infection (Detailed in S7 Table). Of these miRNA families, eleven miRNA families were expressed specifically in *Solanaceae* plants. Among these specific expressed miRNA families, five of them, including miR6022, miR6023, miR6026, miR6027 and miR6024, were predicted to associate with plant innate immune receptors which were listed in Table 3.

**Table 3:** Function description of miRNA families especially presented in the nightshade family (*Solanaceae* plant).

miRNA family	Member	Annotation	Number of Targets	Reference
miR5302	sly-miR5302a, sly-miR5302b-5p, sly-miR5302b-3p	Regulate genes involved in fleshy fruit development	37	[62]
miR5303	sly-miR5303	Regulate genes involved in fleshy fruit development	42	[62]
miR5304	sly-miR5304	Regulate genes involved in fleshy fruit development	1	[62]
miR4376	sly-miR4376	Regulating the expression of an autoinhibited Ca^2+^-ATPase lead to tomato reproductive growth.	1	[63]
miR6022	sly-miR6022	Regulation of plant innate immune receptors.	18	[64]
miR6023	sly-miR6023	Regulation of plant innate immune receptors.	32	[64]
miR6024	sly-miR6024	Regulation of plant innate immune receptors.	48	[64]
miR6026	sly-miR6026	Regulation of plant innate immune receptors.	15	[64]
miR6027	sly-miR6027-5p, sly-miR6027-3p	Regulation of plant innate immune receptors.	29	[64]
miR1919	sly-miR1919a, sly-miR1919b, sly-miR1919c-3p	Not annotated on reference assembly.	4	-
miR9471	sly-miR9471a-5p, sly-miR9471a-3p, sly-miR9471b-5p, sly-miR9471b-3p	Not annotated on reference assembly.	6	-

**Fig 6:**
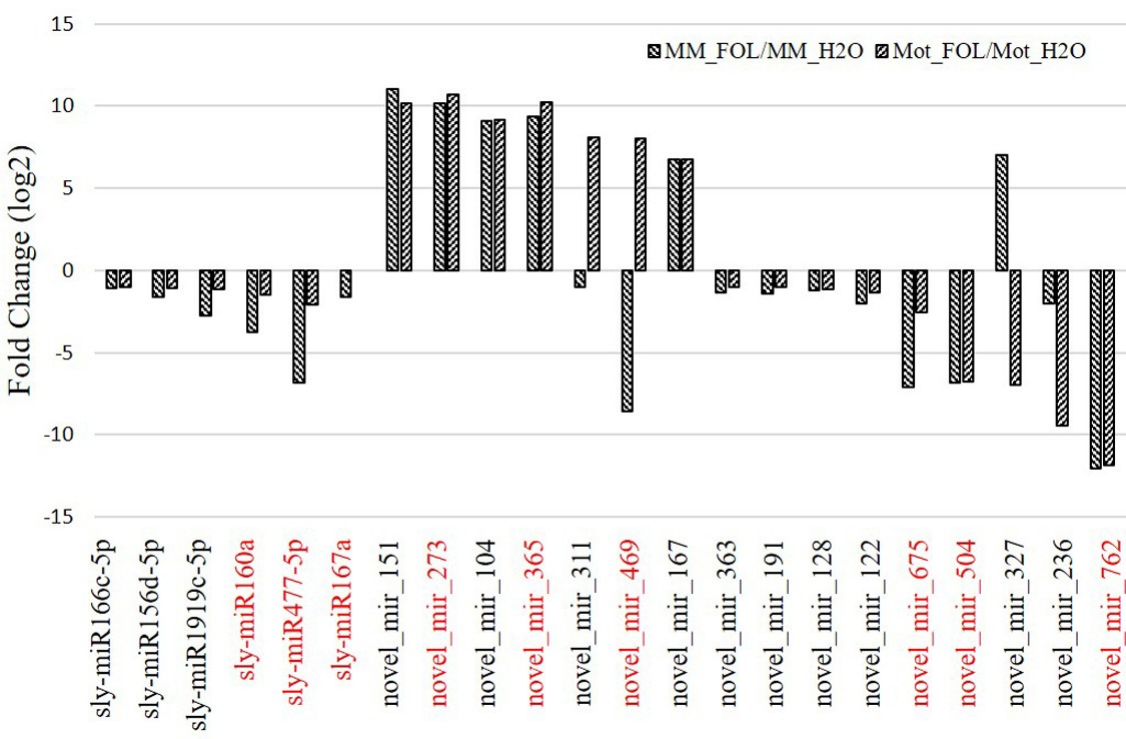
Profiling of miRNAs response to FOL in tomato plants. According to sRNA-seq analysis, partial of regulated miRNAs were summarized by normalizing reads of water treatment for each cultivar.

**Fig 7:**
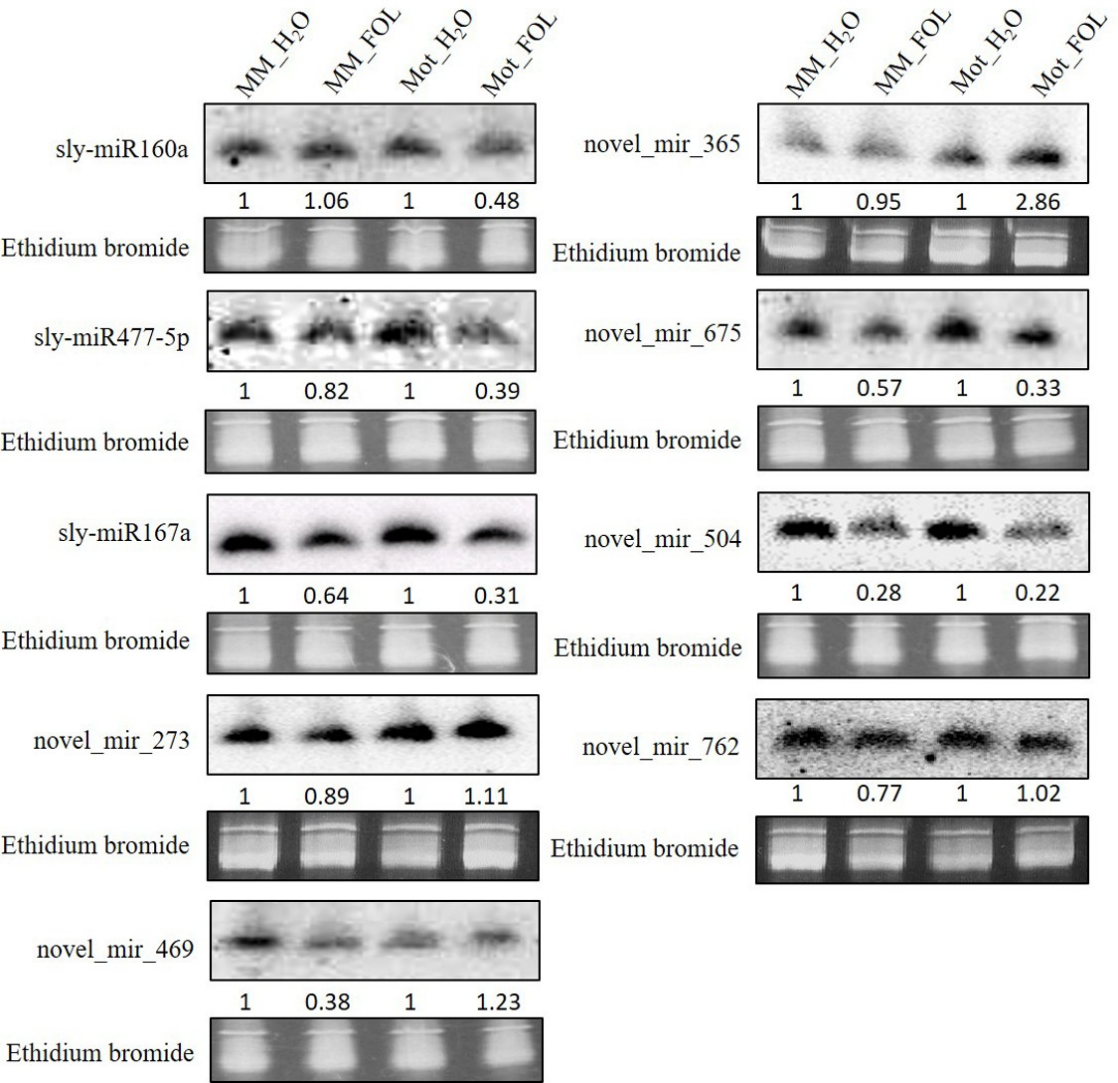
Expression validation of selected miRNAs by Northern blot analysis. MiRNAs including known and novel highlighted with red in Fig 6 were selected randomly for Northern blot analysis. Total root RNA samples (10 μg) were from four treatments. Gel staining with ethidium bromide were used as loading control for each blot. Blots were imaged using a Phosphorimager. Using ImageJ software to measure the grey density, the numbers below each blot present the relative enrichment of individual miRNA in each treatment normalized to the corresponding water-treated control.

To further characterize the miRNAs expression patterns, we identified twenty-two miRNAs up or down-regulated in both Moneymaker and Motelle under FOL/water treatment (Fig 6). To test the reliability of our sRNA-Seq data, Northern blot analysis was performed on nine miRNAs (sly-miR160a, sly-miR477-5p, sly-miR167a, novel_mir_273, novel_mir_469, novel_mir_365, novel_mir_675, novel_mir_504 and novel_mir_762) using primers listed in S8 Table (Detail sequences of novel miRNAs were presented in S9 Table). Our data indicated that sly-miR477-5p, sly-miR167a, novel_mir_675, novel_mir_504 and novel_mir_762 were repressed congruously in both Moneymaker and Motelle when treated with FOL. Novel_mir_365 and novel_mir_469 were slightly up-regulated in Motelle under FOL invasion (Fig 7). These data displayed a credible consistence with the results of sRNA-seq.

## Discussion

In the present study, we explored a global transcriptomic profile of tomato-FOL interaction through four different treatments. The components of plant response to pathogen challenging may lead to understand the potential defense mechanisms. Plants have evolved a complicate defense system against pathogens including cascade signaling activation, the regulation of gene expression, synthesis of defensive metabolites as well as hormone balancing [37, 38]. So far, by taking advantage of high-throughput RNA sequencing (RNA-seq) approach, a few of transcriptome studies discovering the host-FOL interaction have been reported in banana, watermelon, mango and *Arabidopsis* [10–12, 14, 15], shedding light on the cross-talking among different signaling pathways involving in plant-pathogen interaction.

When plant is attacked by pathogen, the host reprograms metabolism balance between development and the resources to support defense to pathogen, involving biological process, cellular components and molecular functions [39]. Based on our results, the tomato-FOL interaction basically follows the typical reaction of necrotrophic pathogens infection. Gene Ontology analysis of DEGs between two tomato cultivars reveals specific enriched categories in both interactions. In resistant tomato cultivar Motelle, cellular component organization or biogenesis, signaling, molecular transducer activity, and signal transducer activity were evidenced when compared to susceptive tomato cultivar Moneymaker. Among them, cellular component organization and biogenesis are critical metabolic activities required by plants to survive under fungus-inflicted stresses [40]. Generally, the genes involved in GO analysis present in Motelle more than in Moneymaker upon FOL infection which may due to different resistant cultivar.

Two main mechanisms, pathogen-associated molecular patterns (PAMPs) [41–43] and the adaptive immune system composed of resistant (*R*) genes [44–46], are involved in plant responses to pathogenic microorganisms in plant. At least five different classes of *R* genes have been classified based on functional domain [46]. Among these classes, a nucleotide-binding site (NBS) and leucine-rich repeats (LRRs) (NBS-LRR) is known as the most numerous *R*-gene class [45]. Previously, we reported that tomato endogenic miRNA slmiR482f and slmiR5300 conferred tomato wilt disease resistance. The targets of both miRNAs were, predicted encoding protein with full or partial NBS domains respectively, confirmed to exhibit function of resistance to FOL [27, 28]. A few of investigations have been demonstrated that NBS-LRR proteins recognize a specific Avr protein and display disease resistance in several plant species, including rice, tomato, *N*. *benthamiana*, *Arabidopsis* and wheat [47–51]. Transcriptional regulation of defense genes has been known as a central in plant defense responses. Certain a few of plant TF families, such as AP2/ERF, bHLH, TGA/bZIP, MYB, NAC and WRKY, appear to be prominent regulators of host defense [52, 53]. Several MYB proteins, including AtMYB30, AtMYB44, AtMYB108/BOSI1 and HvMYB6, demonstrate resistant functions in plant immunity [54]. However, there was no reported MYB protein conferring disease resistance in tomato species.

It is well-established that miRNA is one of the plant produced two major classes of endogenous small RNAs, mediating sequence-dependent post-transcriptional gene silencing (PTGS) by guiding mRNA cleavage and/or translation inhibition. In the past decades, genome-wide small RNA analyses using sRNA-seq approach have been conducted for several plant-filamentous pathogen interactions [27, 55–57]. However, so far, only miR482/2118 superfamily has been elucidated for the disease resistance function in tomato [27, 58]. From our sRNA-seq data, a common set of plant innate immunity miRNAs were regulated after FOL infection in both susceptible and resistant tomato plant. Not surprised, more miRNAs were presented to respond to FOL invasion in Motelle than that of in Moneymaker. Further potential target prediction results by online tool psRNATarget indicated that a few of these targets were pathogen resistant or related genes. For example, sly-miR160a and novel_mir_762 target several Auxin response factors (S10 Table). Auxin is not only an important plant hormone affecting plant development, growth and abiotic stress, but also a new functional molecular to attenuate pathogens virulence [59–61]. Although the high throughput sequencing technology used to characterize the sRNA component did not enable an accurate quantitative evaluation, our bioinformatics analysis combining northern blot confirmed several novel miRNAs conferring FOL infection in tomato, which offered us with an exciting further direction to investigate the role of miRNAs in resistance to FOL in tomato.

To conclude, by integrative analysis, our broad genome transcriptome RNA-seq/sRNA-seq data provide a comprehensive overview of the gene expression profiles of tomato treated with FOL. Our results identified several disease resistance related genes/miRNAs. It will facilitate further analysis of putative molecular mechanism of resistance in tomato leading to the improvement of tomato wilt disease control strategies.

## Supporting information

S1 Table All gene FPKM. (XLSX)

S2 Table GO Classification of mRNA. (XLSX)

S3 Table GO Classification of miRNA. (XLSX)

S4 Table GO pathway enrichment of DEGs_MM. (XLSX)

S5 Table GO pathway enrichment of DEGs_Mot. (XLSX)

S6 Table List of targets of all miRNAs. (XLSX)

S7 Table GO miRNA family analysis. (XLSX)

S8 Table Primer sequences used in this study. (DOC)

S9 Table Targets of verified miRNA. (XLSX)

S10 Table The targets of confirmed miRNAs in this study. (XLSX)
