# Supplementary material for: An integrated analysis of mRNA and sRNA transcriptional profiles in tomato root: Insights on tomato wilt disease
Source: PLoS One. 2018 Nov 5;13(11):e0206765. doi: 10.1371/journal.pone.0206765 (PMC6218063; doi:10.1371/journal.pone.0206765)
Supplement: S8 Table — (DOC) [file pone.0206765.s008.doc]

**Supplementary Table 7 Primers used for qRT-PCR and Northern blot analysis in this study.**

| Gene ID | Application | Sequence (5’ – 3’) | Annotation |
| --- | --- | --- | --- |
| Solyc01g095630 | q-PCR Primer | F: TCCTCATTTGGTGGAGAAGG  R: TAGCTTAGGATCAATTAGGC | WRKY transcription factor |
| Solyc06g068460 | ” | F: GAGTTGGCTAGATTGAGACTG  R: TTGATGCCACAAAAGAGTTG | WRKY transcription factor 1 |
| Solyc03g059080 | ” | F: GCAGTGTGTAGATCCTAAGC  R: CAGTGCCTTGACGACAATTG | Receptor-like protein kinase |
| Solyc03g005570 | ” | F: GATGGAGACATATTGAGGGAC  R: GATACATATATCAAGCATGGC | Myb-related transcription factor |
| Solyc00g174340 | ” | F: ATACTCAAGTAGTCTGGCGC  R: GTAAGGACGTTGTCCGATCC | Pathogenesis-related protein 1b |
| Solyc09g007020 | ” | F: GTGCGGACATTATACTCAAG  R: ACCCAATTGCCTACAGGATC | Pathogenesis-related protein |
| Solyc11g071750 | ” | F: CTTGAAGATGATGTTGAGTCG  R: AAACTAAGAACTCCATCTCC | Calmodulin-like protein |
| Solyc10g006660 | ” | F: ATTAAGTCCTGAGTTGATGG  R: GATAACAGTGCATCAGAAGGG | Calcium-binding EF hand family protein |
| Solyc05g050350 | ” | F: CACAAATGCATCAAGTCTTGG  R: CTAAAATCTGGTTCAGCTGG | Cyclic nucleotide gated channel |
| sly-miR160a | Northern Blot Probe | TGGCATACAGGGAGCCAGGCA | Targeting Auxin response factor gene |
| sly-miR477-5p | ” | GGAGCCCTTGAGGGAGAGACA | Targeting nucleic acid binding protein gene |
| sly-miR167a | ” | TAGATCATGCTGGCAGCTTCA | Targeting multi antimicrobial extrusion gene |
| novel_mir_273 | ” | TGTCGTCCAACCCGACCTCAGA | Targeting Auxin response factor gene |
| novel_mir_469 | ” | ACTTCATTGATCACTAGGCCAC | Targeting receptor serine/threonine kinase gene |
| novel_mir_365 | ” | GATCATCAAAGTTTAATCAGCCG | Targeting receptor like kinase gene |
| novel_mir_675 | ” | AGAACTCAGGATGGATAGCG | None |
| novel_mir_504 | ” | CACATCAGAGAAGAGAGTACCCC | Targeting pentatricopeptide repeat-containing protein gene |
| novel_mir_762 | ” | GGAAGAGCTGAACAATTAATT | Targeting receptor serine/threonine kinase gene |
